# Utility of Aloes in the Treatment of Wounds

**Published:** 1864-10

**Authors:** 


UTILITY OF

### ALOES IN THE TREATMENT OF WOUNDS.

*Formula of an Aloetic Tincture for External Use.*

Aloes is one of the oldest medicines; it enters into a great number of formulas, some of which have been famous for a long time (Elixir de longue vie, Paracelsus Elixir), and others some of which still continue to have a certain amount of credit (Garus's elixir, antecibum pills, etc.). Their number can be conceived on looking over the long list Jourdan gives of them in his *Universal Pharmacopœia*, where, however, the whole of the aloetic formulas are not given. It is, therefore, one of those medicines whose properties are known and appreciated, and about which it would seem there is little to be said. It is scarcely used now-a-days but internally, while it was previously used as much externally as internally. It is not, therefore, useless to recall to the memory of practitioners the uses of this medicine in external therapeutics which have been too much forgotten.

Galen believed aloes applied externally to be an astringent, and stated its properties for closing ulcers. After the Greeks, the Arabs, and a good many therapeuticians who came after them, till the eighteenth century. Geoffrey pointed out aloes as eminently useful in the dressing of wounds and ulcers, and as being susceptible of favoring and quickening their cicatrization, and even of stopping the hemorrhage occasioned by these solutions of continuity. Surgeons in old times used it frequently, eitheir in an alcoholic solution for washing bad ulcers, or as a topic, and mixed with balsamic substances, such as myrrh and incense, in ointments, in balsams, which were used not only in old wounds, but even in recent ones. It entered into the composition of numerous vulneraries, and was thought to prevent suppuration as well as the formation of ulcers, and to promote the adhesion of edges of wounds made by cutting instruments. In these cases the balsam of the Commandeur de Permes, in the composition of which aloes enters, had a great renown. The cut edges were brought together, and over the wound a compress steeped in this compound balsamic tincture was applied. Aloes was also used in composition of collyria for injuries connected with chronic ophthalmia, as well as in injections intended to modify fistulous openings and to promote their obturation.

All these facts seem to be forgotten now. Scarcely a few authors on materia medica mention them, and, if I am not mistaken, there are very few practitioners who now think of the external use of aloes and of the advantages it may present. It is left to veterinary surgery, which had the good sense not to give it up, and which continues to use it with the greatest advantage as vulnerary in the treatment of recent wounds, as well as for modifying and cicatrizing in the dressing of atonic, sanious, fœtid and resisting ulcers.

It is much less, I confess, after reading the old authors than after having been struck with the rapidity with which the aloetic topics cicatrize wounds in animals, that I thought of experimenting on man, I was not long in discovering that with him they were equally useful. The direction of my studies and the nature of my eccupations bring within my reach only a few cases of surgery. I have, however, for a few years got a sufficient number of cases to verify the remarkable cicatrizing properties of aloes. In using the compound medicines in the composition of which it enters, I could not have perfectly appreciated its action. These preparations, more or less complicated, contain balsamic substances, such as myrrh, incense, benzoin, balsams of Tolu or of Peru, which also possess very topic properties that necessarily to a certain extent assist the aloes. I preferred, therefore, using aloes alone, and found it so effectives that in the majority of cases it was not necessary for me to add an adjuvant.

The preparation which I chose for external use is a saturated tincture of alose. The aloetic tinctures were, however, the most valued preparations of the old surgeons. I had, therefore, to support my first trials by the authority of the past; but the formula of these tinctures was very variable, and most of them added at least one other element, balsamical or gommo-resinous. My general formula only contains two elements, alcohol at an ordinary degree of concentration and aloes.

I first of all used one part of aloes to four of alcohol; but I was not long finding out that the more aloes the tincture contained, the more efficaciously it acted on the wounds. I had then to find out in what proportion the alcohol could be saturated with aloes, and I have obtained, with one part of aloes and two of alcohol, a complete solution. On making the dose one part and a half, a deposit is formed which renders useless part of the aloes.

My formula is, therefore, the following: Aloes, 1 part; alcohol, 2 parts. The aloes must be chosen of good quality; Socotrine aloes is the best for external use as well as for internal; hepatic aloes is still valued in veterinary practice really more for its cheapness than for its special properties. As to the impure aloes, called caballin, it must be altogether rejected.

In order to apply the alcoholic tincture of aloes, dip into it a pencil made of lint, and then pass it over the surface of the wounds; or saturate with it the lint used for dressing the wounds. It is evident that this last mode of treatment is more active than the first; therefore it is the one which must be used in the treatment of atonic wounds which show little or no tendency to cicatrize. The application of the tincture of aloes on the wounds is but slightly painful, and even often, as to the sensation there is no local effect.

In the cases in which this treatment has succeeded with me I will mention particularly bed-sores occurring in patients suffering from typhoid diseases or cachectic, and generally so difficult to heal. I get them exclusively dressed with the tincture of aloes, or I have them painted on their surface or around them with this tincture, I then cover them with styrax ointment spread on lint. Bed-sores very seldom resist either one or the other of these treatments. I have also obtained remarkable success in the treatment of old, atonic, and inveterated ulcers; amongst others I will mention two cases of varicose ulcers of the legs, of several years' standing, and which had not been modified by several other applications; having been dressed with perseverance for about two months with lint saturared with tincture of aloes, these ulcers have ended by cicatrizing in an effective manner.

I believe that this topic would be of great advantage in the treatment of the ulcers which follow burns, and which often turn out so badly. In a thousand ways, I may say, surgical therapeutics would rejoice in the use of aloetic topics, and they would deserve to be rescued from their disuse. The formula of tincture which I have just now recommended would be, I think, the best that could be used; a balsamic ingredient such as benzoin or incense could be added in case of its insufficiency.

It will not be without interest to add that we can daily profit by the cicatrizing properties of aloes in the treatment of our domestic animals, in the horse, whose services the doctor so often requires. We all know how much this animal is exposed to sores resulting from blows or knocks which he gets in his stable, or from the rubbing of his collar, his saddle, or other parts of his harness. Now, dressings of the saturated tincture of aloes dry up and heal, with great rapidity, the scratches and sores of horses, and prevent them becoming ulcerated. This lesson in vetrinary surgery should not be despised by city practitioners, and less still by those of the country, who take an interest in the useful and courageous animal companion of their peregrinations.—*Dublin Medical Press*, March 16, 1864, from *Bull. de Thèrap.*

